# Investigation on Super-Resolution Focusing Performance of a TE-Polarized Nanoslit-Based Two-Dimensional Lens

**DOI:** 10.3390/nano10010003

**Published:** 2019-12-18

**Authors:** Yechuan Zhu, Shun Zhou, Zhiheng Wang, Yiting Yu, Weizheng Yuan, Weiguo Liu

**Affiliations:** 1 Shaanxi Province Key Laboratory of Thin Films Technology and Optical Test, Xi’an Technological University, Xi’an 710021, China; zsemail@126.com (S.Z.); wzh_chibakyousuke@163.com (Z.W.); wgliu@163.com (W.L.); 2 Key Laboratory of Micro/Nano Systems for Aerospace, Ministry of Education, Northwestern Polytechnical University, Xi’an 710072, China; yuanwz@nwpu.edu.cn

**Keywords:** super-resolution focusing, nanoslit-based 2D lens, influencing factors, focus length

## Abstract

Conventional optics suffer from the diffraction limit. Our recent work has predicted a nanoslit-based two-dimensional (2D) lens with transverse-electric (TE) polarized design that is capable of realizing the super-resolution focusing of light beyond the diffraction limit in the quasi-far field. Furthermore, the super-resolution capability can be kept in a high-refractive-index dielectric over a wide wavelength range from ultraviolet to visible light. Here, we systematically investigate the influence of various factors on the super-resolution focusing performance of the lens. Factors such as lens aperture, focal length and nanoslit length are considered. In particular, the influence of nanoslit length on lens focusing was ignored in the previous reports about nanoslit-based 2D lenses, since nanoslit length was assumed to be infinite. The numerical results using the finite-difference time-domain (FDTD) method demonstrate that the super-resolution focusing capability of a nanoslit-based 2D lens increases with the lens aperture and reduces with the increase of the lens focal length. On the other hand, it is notable that the length of the lens focus is not equal to but smaller than that of the nanoslits. Therefore, in order to achieve a desired focus length, a lens should be designed with longer nanoslits.

## 1. Introduction

The spatial resolution of conventional optical devices is restricted by diffraction to nearly half the operating wavelength, which greatly limits the performance of all the imaging and focusing systems that lie at the heart of modern biology, electronics and optical integrated circuits [1,2,3]. For more than a century, many efforts have been made to overcome the diffraction limit. In recent years, the interaction between matter such as nanostructures and light has been extensively studied by various research groups, offering possibilities for solving the problem. In 2005, taking advantage of the enhancement of evanescent components of an illuminated object via the excitation of surface plasmon polaritons (SPPs) [4], a superlens built with an ultrathin silver film was developed to achieve imaging that breaks the diffraction limit with a resolution of λ/6 (λ is the operation wavelength) [5]. Moreover, super-resolution focusing was also realized based on metal and dielectric nanostructures [6,7,8,9,10]. These imaging and focusing devices with the capability of overcoming the diffraction limit open great opportunities for the development of a new class of nano-optical devices and techniques. However, the working distance of these devices is greatly confined to the near-field region, or the focus locates inside the structure, which limits the feasibility for actual application.

In order to achieve optical focusing at a greater distance, a large number of planar lenses formed by metallic and dielectric nanostructures with nanostructures used to modulate light have been proposed, such as metallic nanoslits [11,12,13,14,15,16,17], metallic cross-shaped apertures [18], metallic antennas [19], dielectric waveguides [20] and dielectric pillars [21,22,23,24,25,26,27]. Few of these planar lenses could overcome the diffraction limit to realize super-resolution focusing. In 2017, by exciting SPPs and enabling them to couple with radiating propagation modes, a conical two-dimensional (2D) plasmonic-zone plate lens was proposed to realize far-field super-resolution focusing [28]. In our latest research, without the excitation of SPPs, we successfully designed a planar lens composed of metallic nanoslits under the illumination of a TE-polarized plane wave (the polarization parallel to the nanoslit) with a capability of super-resolution optical focusing over a larger distance than twice the operation wavelength [29]. In this article, we extensively explore the influence of various factors on the super-resolution focusing performance of the TE-polarized nanoslit-based 2D lens in order to further improve the method and provide a guide for practical applications. 

## 2. Design and Super-Resolution Focusing of a TE-Polarized Nanoslit-Based 2D Lens

**Figure 1:**
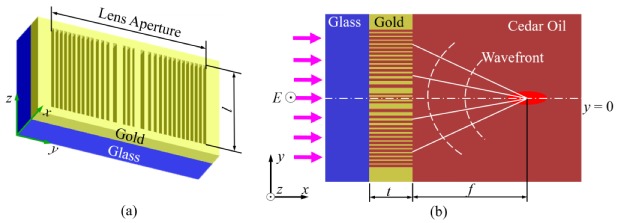
Structure and focusing scheme of a transverse-electric (TE) polarized nanoslit-based two-dimensional (2D) lens. (**a**) The structure of the lens is formed by an array of nanoslits perforated in a gold film located on a glass substrate. (**b**) Schematic focusing of the lens; *t* is the thickness of metal film.

The lens we consider here comprises an array of nanoslits perforated in a gold film on a glass substrate with TE-polarized design [29], which operates in cedar oil under the normal incidence of a plane wave at the wavelength of 405 nm from the glass substrate along the *x* direction, as illustrated in Figure 1. At this wavelength, the relative permittivities of gold and cedar oil are −1.6745 + 5.7286*i* [30] and 2.2952 (*n* = 1.5150), respectively. All the nanoslits have the same length *l*; *t* is the thickness of the gold film, and *f* is the desired focal length.

**Figure 2:**
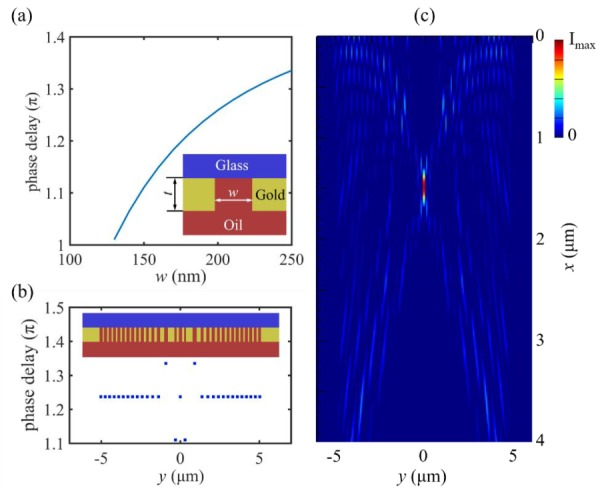
Design and optical focusing of a TE-polarized nanoslit-based 2D lens. (**a**) Phase delay of a 200-nm-deep nanoslit as a function of nanoslit width *w* for the operating wavelength of 405 nm. (**b**) Geometry of the lens (inset) and the phase delay of each nanoslit calculated based on Equation (2). (**c**) Finite-difference time-domain (FDTD) simulated electric field intensity |*E*|^2^ of the lens. The plane *x* = 0 is the exit surface of the lens, and nanoslit length *l* is assumed to be infinite.

In our design, the lens structure is symmetric with respect to central nanoslit at *y* = 0. The basic building unit is a nanoslit surrounded by gold walls, as shown in the inset of Figure 2a. Here, *w* is the width of the nanoslit. The thickness of gold film *t* = 200 nm.

When a TE-polarized plane wave is normally incident on a metallic nanoslit, multiple TE modes can be transmitted through the nanoslit. In order to obtain desired focusing performance, the zeroth mode TE_0_ is considered for the lens design. Therefore, the width of all the nanoslits should be above the cutoff width for TE_0_ mode but below that for the TE_1_ mode, which should meet the following condition:

(1) $$\frac{2\arctan\sqrt{-\varepsilon_{m}/\varepsilon_{d}}}{k\sqrt{\varepsilon_{d}}}<w<\frac{\pi +2\arctan\sqrt{-\varepsilon_{m}/\varepsilon_{d}}}{k\sqrt{\varepsilon_{d}}},$$

where *k* is the free-space wavevector and *ε_m_* and *ε_d_* are the relative permittivities of gold and cedar oil, respectively. The TE_0_ mode of a nanoslit follows the dispersion relationship:

(2) $$\frac{w}{2}\sqrt{\varepsilon_{d}k^{2}-\beta_{0}{}^{2}}=\arctan\frac{\sqrt{\beta_{0}{}^{2}-\varepsilon_{m}k^{2}}}{\sqrt{\varepsilon_{d}k^{2}-\beta_{0}{}^{2}}},$$

where *β*_0_ is the propagation constant for the TE_0_ mode. The above equation links *β*_0_ to the free-space propagation constant *k*, the nanoslit width *w*, and the permittivities *ε_m_* and *ε_d_*. Thus, the phase delay introduced by a nanoslit can be predicted by Re(*β*_0_)*t*. Figure 2a illustrates the phase delay of a nanoslit as a function of nanoslit width. It can clearly be seen that the phase delay strongly depends on nanoslit width. Therefore, we can modulate nanoslit width to control phase delay.

On the other hand, according to the principle of optical interference, the focusing of light at the desired focal distance *f* from a lens can be realized, provided that the phase delay of each nanoslit matches the required phase difference (Δ*φ*(*y*) − Δ*φ*(0)) as a function of the position *y*, which is calculated by:

(3) $$\Delta \varphi (y)-\Delta \varphi (0)=2m\pi +\frac{2\pi n_{d}f}{\lambda }-\frac{2\pi n_{d}\sqrt{f^{2}+y^{2}}}{\lambda },$$

where *λ* is the free-space wavelength, *m* is an arbitrary integer and *n_d_* is the refractive index of the working dielectric for the lens.

Based on Equations (2) and (3), the lens immersed in cedar oil with the desired focal length of 1.5 µm was designed, as shown in the inset of Figure 2b. The geometry of the lens is symmetric with respect to the plane *y* = 0. Beginning from *y* = 0 (the lens center), the position sequence of nanoslits is: 0, 0.304, 0.910, 1.376, 1.746, 2.086, 2.408, 2.718, 3.020, 3.316, 3.606, 3.894, 4.180, 4.462, 4.744 and 5.024 µm. The first three nanoslits have widths of 190, 130 and 250 nm, and all the others are 190 nm. The phase delay of each nanoslit is given in Figure 2b. Nanoslit length *l* is assumed to be infinite.

In order to characterize the focusing performance of the designed lens, full electromagnetic field simulations were performed using a commercial finite-difference time-domain (FDTD) solver (Lumerical Solutions, Inc., Vancouver, BC, Canada). In the simulations, the unit length of FDTD cells was set to 2 nm in both *x* and *y* directions to ensure the convergence of the computation and to model the fine features of the electromagnetic field. Perfectly matched layers as the absorbing boundary conditions were employed around the computational domain. The incident TE-polarized plane wave was defined by setting the electric field component of *E_z_* with the amplitude equaling one.

The simulated electric field intensity pattern of the lens is shown in Figure 2c, which obviously verifies optical focusing behavior. The realized focal length is 1.484 µm (3.664*λ*), demonstrating excellent agreement with the design value. The full width at half maximum (FWHM) of the focus is 98 nm, about *λ*/4.13, well below the diffraction limit (*λ*/(2*n_d_*) = *λ*/3.03 = 134 nm). Therefore, the super-resolution focusing of light in the quasi-far field is achieved. Moreover, the length of the line focus is identical with the nanoslit length, on account that it is assumed to be infinite.

## 3. Effect of Various Factors on the Nanoslit-Based 2D Lens Super-Resolution Focusing

**Figure 3:**
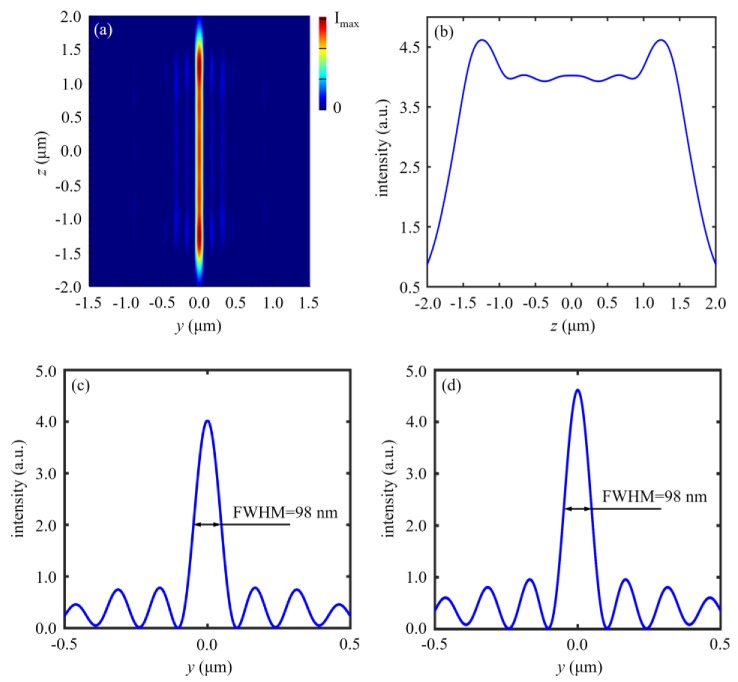
Focusing properties of the TE-polarized nanoslit-based 2D lens with nanoslit length *l* = 4 µm. Simulated electric field intensity |*E*|^2^ distributed (**a**) at the focal plane and (**b**) on the line *y* = 0 in Figure 3a. (**c**) The full width at half maximum (FWHM) of the focus located at the lens center (*z* = 0). (**d**) The FWHM of the focus located at the position of maximum field strength.

We firstly explore the effect of nanoslit length *l* on the focusing properties of the nanoslit-based 2D lens, since the actual nanoslit length cannot be infinite. Figure 3 gives the focusing properties of the TE-polarized nanoslit-based lens with nanoslit length *l* = 4 µm. It can be clearly seen that the length of line focus is approximately 3 µm, namely 75% of the nanoslit length (as shown in Figure 3a). Meanwhile, the maximum field strength is not at the center of the focal plane but near the sides (as shown in Figure 3b). Moreover, the FWHMs of line focus can be kept nearly invariable at different positions, as illustrated in Figure 3c,d.

**Figure 4:**
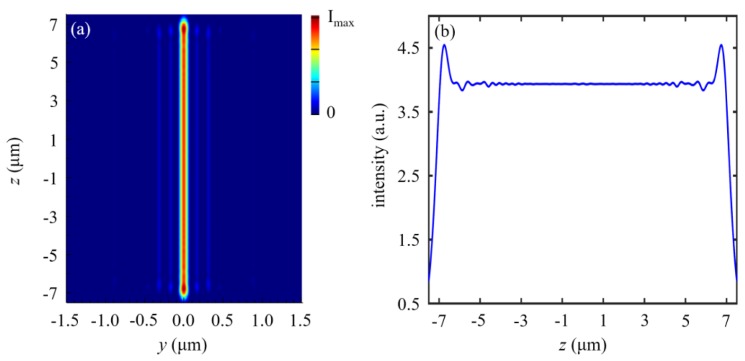
Focusing properties of the TE-polarized nanoslit-based 2D lens with nanoslit length *l* = 15 µm. Simulated electric field intensity |*E*|^2^ distributed (**a**) at the focal plane and (**b**) on the line *y* = 0 in Figure 4a.

Moreover, the length of the line focus approaches the nanoslit length when the nanoslit length becomes larger. Figure 4 shows the focusing properties of the TE-polarized nanoslit-based 2D lens with nanoslit length *l* = 15 µm. The length of the line focus is about 14 µm, namely, 93.33% of the nanoslit length. Because the length of line focus is smaller than the nanoslit length, the real value of the focus length of a nanoslit-based 2D lens should be considered in actual application.

From the above analysis, we can see that the nanoslit length has a significant impact on the focus length of a nanoslit-based 2D lens. On the other hand, the focal size (namely, FWHM) that judges super-resolution capability depends on the aperture and focal length of the lens. Utilizing the same design procedure used in Section 2, a series of nanoslit-based 2D lenses with different apertures was built, which also operate in cedar oil with the designed focal length of 1.5 µm.

**Figure 5:**
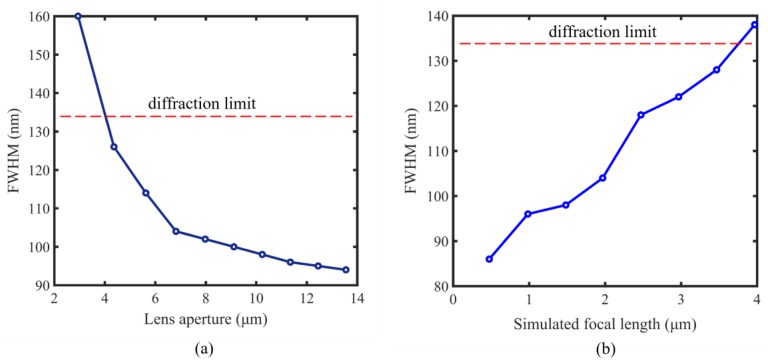
Effect of lens aperture and focal length on the focal size. (**a**) Effect of lens aperture on the lens FWHM. The designed focal length of the lens is 1.5 µm. (**b**) Effect of focal distance on the lens FWHM.

Table 1 illustrates the simulated focusing performances of these lenses. For the lens with a small aperture of 2.942 µm, the real focal length is 1.444 µm. The deviation from the design value is 3.73%, and the FWHM is 160 nm (*λ*/2.53), larger than the diffraction limit of 134 nm (*λ*/3.03). Therefore, for this case, the lens cannot break the diffraction limit. When the lens aperture increases, the super-resolution focusing capability of a lens can be improved and the focal length is also close to the design value, as shown in Table 1. From Figure 5a, it can be clearly observed that the FWHM rapidly decreases and gradually flattens out when the lens aperture increases from 2.942 µm to 13.550 µm.

Focal length also has a great influence on the super-resolution capability of the nanoslit-based 2D lens. Table 2 gives the focusing performance of nanoslit-based lenses with different focal lengths. These lenses were designed with different focal length and nearly identical aperture by using the same design procedure described in Section 2. From Table 2, it can be seen that the FWHM of the lens focus with the focal length of 0.476 µm is 86 nm (*λ*/4.71), much smaller than the diffraction limit of 134 nm (*λ*/3.03), demonstrating an excellent super-resolution focusing ability. However, for the lens with the focal length of 4 µm, the FWHM is 138 nm (*λ*/2.93), larger than the diffraction limit. As a result, the lens cannot break the diffraction limit. The lens with smaller focal length shows better super-resolution focusing ability, as illustrated in Figure 5b.

## 4. Conclusions

In summary, we explore the effect of various factors on the super-resolution focusing properties of a TE-polarized nanoslit-based 2D lens for guiding practical application. The factor of nanoslit length is firstly considered. The results illustrate that the nanoslit length determines the line focus length of a nanoslit-based 2D lens. The latter is less than the former. Nevertheless, the larger nanoslit length can lead to the smaller deviation of the focus length from it. On the other hand, the focal size of a nanoslit-based 2D lens depends on the two parameters, namely, lens aperture and focal length. When the lens aperture is larger than a certain value, the lens can realize super-resolution focusing. Moreover, the super-resolution capability can be improved as the lens aperture increases. The focal length has a decisive impact on the super-resolution capability of a TE-polarized nanoslit-based 2D lens. A smaller focal length can lead to better super-resolution-focusing performance. When focal length is larger than a certain value, the super-resolution focusing will not be achieved, even though the lens has a large aperture. Our work provides a design guide not only for optimizing a TE-polarized nanoslit-based 2D lens but also for optimizing a TM-polarized nanoslit-based lens [11,12,13,14,15,16,17].

## Figures and Tables

**Table 1:** The derived focusing performance of nanoslit-based 2D lenses with different lens sizes.

Lens Aperture (µm)	Designed Focal Length (µm)	Simulated Focal Length (µm)	FWHM (nm)	Max. Intensity (a.u.)
2.942	1.5	1.444	160 (*λ*/2.53)	0.918
4.362		1.472	126 (*λ*/3.21)	1.719
5.626		1.480	114 (*λ*/3.55)	2.264
6.822		1.488	104 (*λ*/3.89)	2.680
7.978		1.488	102 (*λ*/3.97)	3.146
9.114		1.484	100 (*λ*/4.05)	3.677
10.238		1.484	98 (*λ*/4.13)	4.025
11.350		1.484	96 (*λ*/4.22)	4.280
12.454		1.484	95 (*λ*/4.26)	4.607
13.550		1.484	94 (*λ*/4.31)	4.921

**Table 2:** The derived focusing performance of nanoslit-based lenses with different focal lengths.

Designed Focal Length (µm)	Lens Aperture (µm)	Simulated Focal Length (µm)	FWHM (nm)
0.500	10.230	0.476	86 (*λ*/4.71)
1.000	10.564	0.984	96 (*λ*/4.22)
1.500	10.238	1.484	98 (*λ*/4.13)
2.000	10.386	1.968	104 (*λ*/3.89)
2.500	10.482	2.472	118 (*λ*/3.43)
3.000	10.474	2.968	122 (*λ*/3.32)
3.500	10.394	3.468	128 (*λ*/3.12)
4.000	10.230	3.968	138 (*λ*/2.93)
